# Chronic Migraine as a Primary Chronic Pain Syndrome and Recommended Prophylactic Therapeutic Options: A Literature Review

**DOI:** 10.3390/life13030665

**Published:** 2023-02-28

**Authors:** Délia Szok, Anett Csáti, László Vécsei, János Tajti

**Affiliations:** 1 Department of Neurology, Albert Szent-Györgyi Medical School, University of Szeged, Semmelweis u. 6, H-6725 Szeged, Hungary; 2 ELKH-SZTE Neuroscience Research Group, Semmelweis u. 6, H-6725 Szeged, Hungary

**Keywords:** chronic, migraine, pain, primary, prophylactic, real-world evidence, treatment

## Abstract

Chronic pain conditions have a high socio-economic impact and represent a burden for patients, and their management is a challenge for healthcare professionals. Chronic migraine is one of the chronic primary headache disorders, which belong to chronic primary pain syndromes as per the new concept of multiple parenting. The aims of this review were to provide an overview of the latest classification systems involving both entities, the epidemiological data, and the currently recommended prophylactic treatment options for chronic migraine. Randomized controlled clinical trials, meta-analyses, real-world data, and review articles were analyzed. Chronic migraine is a prevalent and highly burdensome disease and is associated with high headache-related disability and worsening health-related quality of life. Treatment of chronic migraine includes pharmacological or, in drug-refractory cases, non-pharmacological (e.g., neuromodulatory) approaches. Among pharmacological treatment options, injectable botulinum toxin type A and calcitonin gene-related peptide-targeting human and fully humanized monoclonal antibodies (i.e., eptinezumab, erenumab, fremanezumab, and galcanezumab) are highly recommended in the preventive treatment of chronic migraine. Novel migraine-specific therapies offer a solution for this devastating and difficult-to-treat chronic pain condition.

## 1. Introduction

Chronic migraine (CM) is listed in both the headache and the chronic pain sections in the recent classification system of the International Association for the Study of Pain (IASP) [1]. Migraine is one of the primary headache disorders with well-defined subclasses. The main subtypes are migraine without or with aura. Both forms can be episodic or chronic. The latest version of the International Headache Society classification, the International Classification of Headache Disorders 3rd edition (ICHD-3), was the first to recognize CM as a distinct entity, representing a subclass of migraine (Table 1). The term chronic means that the patient has 15 or more headache days per month for more than 3 consecutive months, with at least 8 out of the 15 head pain episodes showing features of migraine without or with aura [2]. In episodic migraine (EM), the headache days are lower than 15 days per month. In clinical studies, two subclasses of EM are used: low-frequency EM with 4–8 headache days per month, and high-frequency EM with 9–14 headache days per month.

**Figure 1:**
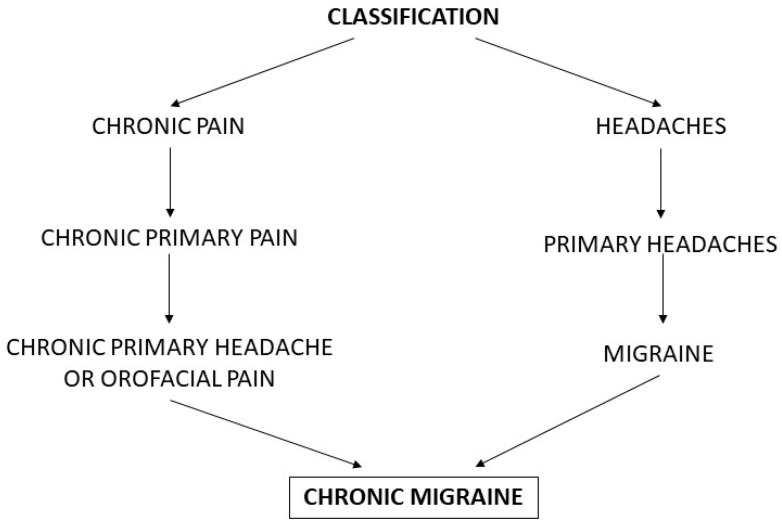
Classification systems for chronic migraine.

CM is also one of the chronic primary pain syndromes [3]. The latest classification of chronic pain announced by the IASP distinguishes between primary and secondary chronic pain conditions. Among chronic primary pain syndromes, CM belongs to the chronic primary headache or orofacial pain subclass (Figure 1) [3].

CM is a prevalent and highly burdensome disease. The prevalence of CM is much lower than that of episodic migraine (EM) (1.4–2.2% versus 14.4%) in the general population, but CM is associated with a greater headache-related disability and lower health-related quality of life compared to EM [4,5].

At present, among the most effective prophylactic pharmacotherapies of CM are botulinum toxin type A (BoNT-A) and human and fully humanized calcitonin gene-related peptide (CGRP)-targeting monoclonal antibodies (mAb). In drug-refractory, difficult-to-treat CM patients, neuromodulatory techniques may provide a rescue in therapeutic options.

This literature review was conducted to discuss the role of the different classification systems in establishing the proper diagnosis of CM, to provide an overview of the recent epidemiological data and the economic burden of CM, and to summarize the clinical and scientific background of the recommended CM-specific preventive treatment options.

## 2. Materials and Methods

The literature analysis included randomized controlled clinical trials (RCTs), meta-analyses, real-world data, and review articles published in English, focusing on works published since the date when CM was first classified as a distinct entity in the ICHD-3beta in 2013. Earlier articles were discussed only if they contained crucial information regarding the topic with no temporal limitations. The electronic literature search was conducted using the PubMed database up to November 2022, by using multiple combinations of keywords such as ‘botulinumtoxin type A’, ‘CGRP’, ‘chronic’, ‘migraine’, ‘monoclonal antibody’, ‘neuromodulation’, ‘onabotulinumtoxinA’, ‘pain’, ‘primary’, ‘preventive’, ‘preventative’, ‘prophylactic’, ‘syndrome’, ‘real-world evidence’, ‘therapy’, and ‘treatment’.

## 3. Results

### 3.1. Classification of CM as a Chronic Primary Pain

The latest definition of pain released by the IASP in 2020 is the following: ‘An unpleasant sensory and emotional experience associated with, or resembling that associated with, actual or potential tissue damage’ [6], whereas ‘chronic pain is pain that persists or recurs for longer than three months’ [1]. Chronic primary pain is multifactorial, with biological, psychological, and social factors contributing to the development of the pain syndrome [3].

The subclasses of chronic primary pain are chronic widespread pain (e.g., fibromyalgia), complex regional pain syndrome, chronic primary headache (including CM, chronic tension-type headache, and chronic trigeminal autonomic cephalalgias (including chronic cluster headache)) or orofacial pain (e.g., chronic temporomandibular joint pain), chronic primary visceral pain (e.g., chronic irritable bowel syndrome), and chronic primary musculoskeletal pain (e.g., chronic non-specific low back pain) (Table 2) [1,3]. The diagnostic criteria of chronic primary pain include (1) being persistent or recurrent for longer than 3 months, (2) being associated with emotional distress (e.g., anxiety, anger/frustration, or depressed mood) or functional disability (e.g., interference with daily life activities and social participation), and (3) not being better accounted for by another diagnosis (Table 3) [3].

Chronic primary headache or orofacial pain as a subclass of chronic primary pain is defined as a headache or orofacial pain that occurs on at least 15 days per month for longer than 3 consecutive months. The duration of pain per a headache day is at least 2 h (if untreated), or it may present in several shorter attacks per day [3]. By definition, in CM, a headache occurs on 15 or more days per month for more than 3 consecutive months, which on at least 8 days per month has features of migraine with or without aura [2]. The description of migraine involves recurrent head pain attacks lasting for 4–72 h usually with a unilateral location and a pulsating quality, but pain may be bilateral or tightening, moderate or severe in intensity, aggravated by routine physical activity, and associated with nausea and/or photophobia and phonophobia [2]. The diagnostic criteria of CM are presented in Table 4.

### 3.2. Epidemiology of CM

The prevalence of CM is 1.4–2.2% in the general population, whereas it is present in 7.7% of migraine patients [7]. Chronification of EM to CM occurs in about 3% of EM patients per year [8]. The incidence of CM is difficult to establish, with a survey declaring a 2.5% annual incidence for CM [9].

The CM Epidemiology and Outcomes (CaMEO), a prospective, cross-sectional and longitudinal, web-based cohort study, involved a huge number of migraine patients and was designed to characterize the course of CM and EM [10]. The main domains addressed by this study were headache frequency, headache-related disability, comorbidity, medication use, and familial impact. An analysis of this study assessed the barriers to successful care of CM, which are the lack of adequate medical consultation, lack of diagnosis, and lack of treatment. This analysis revealed that less than 5% of CM patients passed the above three barriers to receive adequate care for headache [11]. A further study continuing these aspects concluded that only 8% of migraineurs traversed all four analyzed barriers to care (i.e., the lack of consultation, lack of diagnosis, lack of pharmacological treatment, and lack of avoidance of medication overuse), with a rate of only 1.8% in CM [12]. Another analysis of the CaMEO study compared the data of an American Migraine Prevalence and Prevention (AMPP) study regarding demographic characteristics, headache features, and disability (measured by Migraine Disability Assessment (MIDAS)). Both studies were conducted in the USA and were concordant in finding more severe headache-related disability in CM patients compared to EM patients [13]. An analysis of the CaMEO study addressing the effects of coexistent noncephalic pain (such as that affecting the face, neck/shoulders, back, arms/hands, legs/feet, chest, or abdomen/pelvis) in migraineurs revealed an associated increased risk for migraine chronification [14]. Similarly, low back pain as a noncephalic chronic pain condition has been reported to elevate the risk of primary headache chronification [15]. 

The AMPP study (a longitudinal, population-based survey) analyzed the rates of common comorbidities associated with CM. Chronic musculoskeletal pain was found to be 2.49 times more frequent in CM than in EM patients [16]. Chronic overlapping noncephalic pain conditions (COPCs) such as temporomandibular disorder, back pain, fibromyalgia, irritable bowel syndrome, and endometriosis can be linked to migraine, and patients with concomitant CM and COPCs form a distinct subgroup of CM. A cross-sectional retrospective observational study (Collaborative Health Outcomes Information Registry (CHOIR)) revealed that this subgroup of patients is associated with significantly worse pain-related physical and psychosocial functions as well as greater healthcare utilization compared to CM-alone patients [17]. Focusing on important life domains (such as marital, parenting, romantic, and familial relationships and career-related/financial success), an analysis of the CaMEO study observed a negative impact of migraine on these domains, and the burden was greater in CM patients compared to EM patients [18]. Analyzing the data from the CaMEO study regarding the frequency of acute medication overuse and its effect on headache burden among EM and CM patients revealed that migraineurs with medication overuse had a greater disease burden and increased urgent care use compared to those without medication overuse [19].

Based on the AMPP study, the yearly transition rate from EM to CM is estimated to be between 2.5% and 3% [7]. Risk factors for the conversion from EM to CM include modifiable or non-modifiable ones, and their identification and elimination are crucial in the management of CM patients [8]. Risk factors for the chronification of migraine with strong/moderate evidence are headache day frequency, depression, obesity, ineffective acute treatment, and acute medication use/overuse [7,8]. Patients suffering from CM face a high risk of developing medication overuse headache (MOH), which significantly impairs their quality of life.

### 3.3. Socio-Economic Impact—The Burden of CM

CM represents a major economic burden for society [20]. An observational study performed in an Italian tertiary-level headache center pointed out that the annual direct cost of CM was higher (2037 Euro) than that of EM (427 Euro), which is a 4.8-fold difference [21]. In this study, the mean annual cost of acute medication was 191 Euro in CM patients compared to 123 Euro in EM patients. [21].

The International Burden of Migraine Study conducted in the USA and Canada concluded that CM was associated with higher total mean headache-related costs compared to EM (in the USA, 1036 USD in CM versus 383 USD in EM, and in Canada, 471 Canadian dollars in CM versus 172 Canadian dollars in EM [22].

### 3.4. Treatment of CM

Treating chronic pain conditions such as CM is always challenging for healthcare professionals. The complex mechanisms that lead to the chronification of pain require a multidisciplinary approach and multimodal therapeutic management.

The treatment of CM follows the guidelines of EM, which involves pharmacological (acute and prophylactic) and non-pharmacological therapeutic options (e.g., psychotherapy and neuromodulation) [8,20,23]. The acute pharmacological treatment of CM is similar to that of EM, including non-steroidal anti-inflammatory drugs, triptans, and gepants. In the last decade, BoNT-A injections have been recognized as an efficacious and justified prophylactic treatment for CM. In recent years, however, CGRP-targeting monoclonal antibodies (mAbs) have become new players in this field. These substances can be considered real game changers. On the one hand, they reframed the hypothesis of the possible sites of action of prophylactic drugs for CM (i.e., the part of the trigeminovascular system (TS) that is outside the BBB). On the other hand, not surprisingly, they became one of the strongly recommended therapeutic options in the prophylactic treatment of CM.

In drug-refractory CM cases, neuromodulatory techniques (i.e., non-invasive/transcutaneous or invasive/implanted methods, which can target either the peripheral or the central nervous system) can provide pain relief [4,8,20,23].

## 4. Recommended Prophylactic Treatment Options in CM

### 4.1. BoNT-A Injection for the Treatment of CM

Emil Pierre-Marie van Ermengem was the bacteriologist who isolated *Clostridium botulinum* in 1895. The botulinum neurotoxins (seven serotypes from A to G) are members of the clostridial neurotoxin family [24]. Concerning CM therapy, the BoNT-A serotype is of therapeutic use. Early observations suggested an analgesic effect of BoNT-A in patients suffering from dystonia. After receiving a BoNT-A injection, the patients reported pain relief before experiencing muscle tone reduction [25]. Another intriguing real-life observation was that BoNT-A used for cosmetic purposes (such as to treat facial wrinkles) decreased the headache frequency in migraineurs [26]. Preclinical studies demonstrated that BoNT-A inhibited the evoked (i.e., potassium chloride- or capsaicin-induced) release of CGRP in primary cultures of rat trigeminal ganglia [27]. This effect is based on the cleavage of the soluble N-ethylmaleimide-sensitive factor attachment protein receptor (SNARE) complex, thereby diminishing the function of synaptosomal-associated protein of 25 kDa (SNAP-25) [24]. Preclinical studies demonstrated that BoNT-A could act on the primary sensory (first-order) neurons. BoNT-A could reverse the sensitization of the C-meningeal nociceptors, and moreover diminish the release of inflammatory mediators and neurotransmitters (such as substance P, CGRP) in the trigeminal ganglia. BoNT-A via the supposed modification of the antidromic influence from the dura to the brainstem, thalamus, and cortical neurons can decrease central sensitization and influence cortical excitability. BoNT-A has the capability to change phenomena due to central sensitization like photophobia or multisensory integration [28,29]. 

Clinical observations demonstrated that BoNT-A (a.k.a. onabotulinumtoxinA) therapy decreased the interictal CGRP levels in the plasma of CM patients. Interestingly, this effect was realized only in the BoNT-A responder CM patient population [30]. After these fundamental observations, two RCTs, the Phase III Research Evaluating Migraine Prophylaxis Therapy (PREEMPT)-1 and PREEMPT-2 were conducted, which were followed by the publication of the analysis of the pooled results from the PREEMPT clinical program [31,32,33]. The pooled results showed a significant decrease in the mean change from baseline in frequency of headache days as a primary endpoint (BoNT-A: −8.4 days; placebo: −6.6 days; *p* value < 0.001) at week 24. The most commonly reported AEs were neck pain (6.7%), localized muscular weakness (5.5%), and eyelid ptosis (3.3%) (Table 5) [33]. These results and the applied treatment paradigms led to the development of the methodological recommendations in the form of a fixed-site (i.e., 31–39 sites including the corrugator, procerus, frontalis, temporalis, occipitalis, cervical paraspinal, and trapezius muscle injection sites) and fixed-dose (i.e., 155–195 units) regimen every 12 weeks for up to 5 treatment cycles [34]. A Cochrane meta-analysis concluded that BoNT-A reduced the number of monthly migraine days (MMDs) by 2 days compared to placebo in the preventive treatment of CM patients [35]. A meta-analysis of 10 years of real-world data demonstrated that BoNT-A diminished the number of monthly headache days (MHDs) and the number of days of acute headache pain medication intake per month, improved the total Headache Impact Test (HIT)-6 score, the MIDAS score, and the Migraine-Specific Quality of Life score, and increased the ≥50% reduction in migraine days response rate [36]. A recent analysis of available CM preventive treatments (i.e., oral topiramate, anti-CGRP mAbs, and onabotulinumtoxinA) concluded that, to date, only onabotulinumtoxinA had long-term safety data in real-world settings, reporting AEs of up to 3 years [37]. A systematic review focusing on the economic evaluation of preventive pharmacotherapeutic options for CM patients revealed that BoNT-A was cost-effective compared to placebo, with an incremental cost-effectiveness ratio ranging between 17,720 and 19,572 Euro [38].

CM is associated with several psychiatric comorbidities such as depression and anxiety. A prospective, open-label, multicenter, pilot study evaluated not only the efficacy and safety of prophylactic BoNT-A treatment but also the comorbid depressive symptoms based on different questionnaires in 32 CM patients with depression. The results showed statistically significant improvements in Beck Depression Inventory-II (BDI-II) (−7.9, *p* < 0.0001), Patient Health Questionnaire depression module (−4.3, *p* < 0.0001), and Generalized Anxiety Disorder questionnaire (−3.5, *p* = 0.0002) scores [39]. A clinical trial evaluating the effect of BoNT-A on efficacy, depression, anxiety and disability using MIDAS, BDI, and Beck Anxiety Inventory (BAI) in patients with CM concluded that a BDI score at 3 months of treatment was 7.67 ± 4.63 compared to the baseline (without treatment (16.13 ± 9.29) (*p* < 0.041)). The BAI scores showed 12.78 + 8.35 in the third month of the treatment compared to baseline (13.81 ± 9.22) (*p* = 0.8) [40]. 

In conclusion, BoNT-A treatment is confirmed to be safe, well-tolerated, effective, and presents an evidence-based option in the preventive treatment of CM patients [41,42,43,44,45,46].

### 4.2. CGRP-Targeting mAbs for the Treatment of CM

CGRP is a 37-amino acid-containing neuropeptide, which has a pivotal role in the activation mechanism of the TS. CGRP has two isoforms, alpha- and beta-CGRP. Alpha-CGRP is expressed in the peripheral and central nervous systems, while beta-CGRP is predominantly present in the enteric nervous system. The landmark finding that shed light on the role of CGRP in the pathomechanism of migraine was a clinical observation that the plasma level of CGRP was elevated in the external jugular venous cranial outflow in migraineurs during the ictal phase [47]. Another interesting clinical finding showed that the human alpha-CGRP infusion resulted in delayed migraine-like headache in migraine patients [48]. Fine immunohistochemical research revealed that the pseudounipolar neurons in the human trigeminal ganglia contain CGRP and CGRP receptor elements such as receptor activity-modifying protein 1 (RAMP-1) and calcitonin-receptor-like receptor (CRLR). However, the satellite glial cells contain only RAMP-1 and CLRL [49,50,51,52,53].

The exact pathomechanism of migraine is still poorly understood; however, the most widely accepted hypothesis is related to the activation of the TS [54]. The skeleton of the TS is the bridge between the meninges, the cortical and meningeal vasculature, and the second-order nociceptive neurons in the trigemino-cervical complex, and this bridge is formed by the peripheral and central branches of the first-order nociceptive neurons in the trigeminal ganglion [54,55,56]. The second-order neurons convey the information to the thalamus (i.e., third-order neurons) which is finally transferred to the somatosensory cortex. The characteristic clinical sign of central sensitization is cutaneous allodynia, which refers to an abnormal painful sensation elicited by a stimulus that does not normally provoke pain. The site of allodynia reflects the localization of the hyperexcited neurons. Allodynia of the face and neck (i.e., cephalic allodynia) is the consequence of the hyperexcitation of second-order neurons in the trigemino-cervical complex, whereas allodynia on the arm (i.e., extracephalic allodynia) is related to the overactivation of third-order neurons in the thalamus [57]. The function of the second-order neurons is influenced by the periaqueductal grey matter, the nucleus raphe magnus, and the locus coeruleus [58,59]. 

Recent neuroimaging studies with different modalities have provided insights into the pathophysiology of CM regarding the involvement of distinct migraine-related brain structures. Positron emission tomography (PET) studies showed abnormal energy metabolism in the upper brainstem (dorsal rostral pons); magnetic resonance spectroscopy (MRS) studies demonstrated dysfunction in the thalamocortical tract, whereas functional MRI studies showed alterations in the activity of the hypothalamus [60]. The function of the TS is to manage peripheral and central sensitization and hyperexcitation during a migraine attack [20,61,62,63]. 

Modern and innovative pharmaceutical techniques lead to the development of fully humanized and human mAbs against CGRP (i.e., fremanezumab, galcanezumab, and eptinezumab) or CGRP receptor elements (i.e., erenumab). These agents create a new era in the prophylactic treatment of EM and CM [64,65]. The mAbs are huge molecules with a consequent near-complete inability to pass the BBB; therefore, these substances target structures outside the BBB, such as the trigeminal ganglia and cerebral dura mater [64,66,67]. For the purpose of good clinical practice, the principal aim is to verify the efficacy, safety, and tolerability of a drug via well-designed RCTs, meta-analyses, and long-term, real-world studies.

In the following sections, the most prominent findings with anti-CGRP mAbs in the treatment of CM are summarized (Table 6).

#### 4.2.1. Erenumab

Erenumab is a human mAb (IgG2alpha), which is the only one in this group of medications that targets the CGRP receptor. At present, the route of its administration is subcutaneous (SC) because an oral formulation is not yet available.

A phase 2 RCT analyzing the efficacy and safety of SC (self-injectable) erenumab in doses of 70 mg or 140 mg given every 4 weeks for 12 weeks in CM patients demonstrated that the MMDs significantly decreased in the treatment group (−6.6 days) compared to the placebo group (−4.2 days) (*p* < 0.0001). The most common AEs were nasopharyngitis (70 mg 3%; 140 mg 2%; placebo 6%), constipation (70 mg 0%; 140 mg 4%; placebo 1%), and injection-site pain (70 mg 4%; 140 mg 4%; placebo 1%). The safety profile of erenumab was similar to that of the placebo [70].

A real-world, long-term (52-week), single-center, prospective, observational study evaluating the efficacy and safety of erenumab in adult CM patients revealed effectiveness and good tolerability [73].

Mutual expectations of patients and healthcare professionals include the rapid effect of a drug either in an acute or in a prophylactic setting. In this context, it should be emphasized that both doses of erenumab showed an early onset of efficacy (within the first week of treatment), with significant differences in the mean change from the baseline in weekly migraine days (at 70 mg: −0.9 days and at 140 mg: −0.8 days) compared to the placebo (−0.5 days) in CM patients [74].

Modern clinical medicine does not only focus on the evaluation of the efficacy and safety of a drug, but it also assesses factors which influence the everyday life of the patient. 

An RCT analyzing the effect of erenumab on patient-reported outcomes such as health-related quality of life, headache impact, and disability in CM observed relevant improvements in all domains at both doses of erenumab (70 mg and 140 mg) at 3 months [75].

Overuse of pain killers taken for headaches can lead to the development of an independent disease entity referred to as MOH. A subgroup analysis of an RCT evaluating the effect of erenumab in CM patients with or without medication overuse revealed similar effects regarding MMDs in both subgroups [76]. 

Notably, a prospective, single-center, real-world audit analyzing the effectiveness and tolerability of erenumab in medically refractory CM with and without medication overuse revealed that the percentage of patients with medication overuse was reduced from 54% at baseline to 20% at month 3 and to 25% at month 6 [77].

A prospective, single-center, real-life cohort study focusing on highly resistant CM patients (i.e., CM with MOH, where all patients had previously failed to respond to BoNT-A and to at least 10 pharmacological and non-pharmacological preventive therapies in total) observed that erenumab reduced the median MMDs from baseline from 26.0 to 13.0 days [78]. 

An Italian single-center, real-life subgroup analysis revealed that two-thirds of CM patients converted to EM during a 6-month erenumab therapy (median MHDs decreased from baseline 26.5 to 7.5 days) [79].

A post-hoc analysis of a 12-week RCT and a 52-week open-label extension study evaluating the effect of erenumab in promoting reversion from CM to EM demonstrated that 54.1% of the patients converted to EM within 12 weeks, whereas a delayed reversion to EM was observed in 43.4% of patients who remained in CM at week 12. This benefit was dose-dependent (with higher reversion rate at 140 mg than at 70 mg) [57]. 

It is of debate whether the presence of cutaneous allodynia can influence the efficacy of erenumab, similarly to its influence on the efficacy of triptans. A post-hoc subgroup analysis of a 12-week-long RCT revealed that CM patients with and without ictal allodynia responded to erenumab treatment in the same manner, allowing the conclusion that the presence of allodynia had no impact on the effectiveness of erenumab [57].

Current therapeutic guidelines recommend CGRP-targeting mAb treatment for up to 1 year for CM patients. A multicenter, prospective, real-world-evidence life study revealed a significant worsening in MMDs 3 months after the discontinuation of erenumab in patients with CM and MOH at baseline [80].

Although migraines predominantly affect young and middle-aged populations, it can still occur in the elderly, with associated comorbidities. A pooled and age-stratified analysis of the safety data of erenumab in CM concluded that erenumab was well tolerated and safe in patients over 60 years old [81].

It is still unclear whether the effectiveness of mAb treatment is long-lasting in CM patients. Addressing this issue, a 2-year-long, real-world, prospective analysis was conducted in resistant CM patients who failed more than three migraine prophylactic medications. During this 2-year-long period, the sustained effectiveness of erenumab was reported only in a small proportion of patients. The rate of 50% responders (i.e., with 50% reduction in their mean MMDs) to erenumab was 31% at 6 months, 26% at 12 months, 24% at 18 months, and 16% at 24 months [82].

Scenarios where recommended evidence-based treatments are not effective, not tolerated, contraindicated, or financially inaccessible for a patient represent a great challenge for healthcare professionals. A real-world study found that onabotulinumtoxinA-resistant CM patients showed improvements in pain, acute drug use, and quality of life following erenumab treatment [83]. 

In addition, comparing erenumab with onabotulinumtoxinA as preventive treatments in CM suggested directional benefits (without reaching the threshold of statistical significance) in MMDs and MHDs favoring erenumab versus onabotulinumtoxinA [84].

A prospective observational cohort study assessing the effect of erenumab on efficacy and quality of life parameters (mental well-being, anxiety, and depression levels) examined with the validated Migraine-Specific Quality-of-Life Questionnaire—Version 2.1 (MSQ V2.1) concluded that in the 3-month-interval treatment period, 81.3% of the patients reported an improvement in the examined parameters [85]. 

Based on the results from well-designed RCTs and data obtained from meta-analyses and real-life studies, it can be concluded that the erenumab SC monthly treatment is effective, safe, and well-tolerated in CM patients.

#### 4.2.2. Fremanezumab

Fremanezumab is a fully humanized mAb (IgG2) targeting the CGRP ligand, administered in form of a SC injection (self-injectable). A 12-week phase 3 RCT studied fremanezumab for the preventive treatment of CM in two dose regimens (quarterly: a single dose of 675 mg at baseline and placebo at weeks 4 and 6; monthly: 675 mg at baseline and 225 mg at weeks 4 and 8). The least-squares mean reductions in the average number of MHDs (i.e., the primary endpoint of this study) was 4.3 in the quarterly regimen, 4.6 in the monthly regimen, and 2.5 in the placebo group, with the differences being statistically significant for both doses compared to placebo (*p* < 0.001) [71]. Nasopharyngitis (fremanezumab quarterly 5%, monthly 4%, placebo 5%) and injection site pain (fremanezumab quarterly 30%, monthly 26%, placebo 28%) were the most frequent AEs associated with fremanezumab [71].

The scale of the effectiveness of fremanezumab is well reflected by the phase 3b FOCUS study, which examined EM and CM patients with documented previous failure in up to four migraine preventive medication classes, and demonstrated favorable results in this difficult-to-treat subgroup of migraineurs [86]. The open-label extension of this study revealed improvements in quality of life, depression, and work productivity achieved during a 6-month treatment period [87].

The HALO CM study was designed to analyze the effect of fremanezumab on quality of life, productivity, and overall health status in CM patients and concluded that the fremanezumab treatment resulted in improvement in all examined parameters compared to the placebo [88]. A subgroup analysis of the HALO-CM study focusing on the effects of fremanezumab concluded there was significant treatment benefit over placebo in patients with CM and comorbid moderate to severe depression. LSM change from baseline in the fremanezumab administered quarterly group was −5.4 days per month; in the fremanezumab administered monthly group, it was −5.5 days per month; and in the placebo group, it was −2.4 days per month. Direct comparisons between fremanezumab quarterly and placebo arms were −3.1 days/month (*p* < 0.001), and fremanezumab monthly and placebo −3.0 dyas/month (*p* = 0.002) [89].

From the real-world aspect, a retrospective, panel-based, online chart review analyzed the effectiveness of fremanezumab among CM patients in the US and demonstrated that the mean reduction from baseline in MMDs at month 6 was 10.1 [90].

Regarding safety, a pooled analysis of three 12-week phase 3 RCTs evaluating the safety and tolerability of fremanezumab in CM patients revealed favorable overall and cardio- and cerebrovascular safety profiles [91]. The pooled analysis of HALO-EM, HALO-CM, and FOCUS studies evaluating EM and CM patients aged ≥60 years revealed that fremanezumab is efficacious and well-tolerated even in this subgroup of migraineurs [92]. The FRIEND study, a 12-week, multi-center, prospective, real-life, cohort study focusing on the effectivity, safety, and tolerability profiles of fremanezumab in the preventive treatment of CM patients confirmed the beneficial features of the drug; in fact, the effectiveness of fremanezumab was higher in the real-life setting than in the RCTs [93].

A pooled analysis of the HALO-EM, HALO-CM, and FOCUS clinical trials showed obvious improvements in disability severity in this patient population [94].

Overall, monthly or quarterly SC administration of fremanezumab is effective, safe, and well tolerated, and it greatly improves the health-related quality of life of CM patients in long-term and real-world conditions.

#### 4.2.3. Galcanezumab

Galcanezumab is a fully humanized mAb (IgG4) targeted against the CGRP ligand and is used for EM and CM administered in form of a SC injection (self-injectable).

In a phase 3 RCT (REGAIN study), galcanezumab in both dose regimens (240 mg loading dose followed by 120 mg monthly, or 240 mg monthly from the beginning to the end of the treatment period) reduced the mean MMDs across the 12-week treatment period compared to the placebo for the preventive treatment of CM (placebo: −2.7; galcanezumab 120 mg: −4.8, galcanezumab 240 mg: −4.6 least-squares mean; *p* < 0.001). The common AEs were nasopharyngitis (120 mg 6%; 240 mg 3%; placebo 5%) and injection-site pain (120 mg 6%; 240 mg 7%; placebo 4%) [72]. 

The post-hoc analysis of the REGAIN study concerning CM patients with a medical history of anxiety and/or depression revealed that anxiety and/or depression had significant reductions in overall MHD frequency with the 240-mg dose (mean change difference from placebo 95% CI: −1.92, *p* = 0.018), but not with the 120-mg dose of galcanezumab [95].

The long-term, open-label extension phase of the REGAIN study during the 9-month period showed reduction in MMDs (at baseline: 19.4; placebo: −8.5; 120 mg galcanezumab: −9.0 and 240 mg galcanezumab −8.0) [96].

A phase 3b multicenter RCT with galcanezumab (CONQUER study) was designed for migraine patients who previously failed from two to four standard-of-care preventive medications of EM and CM patients aged 18–75 years with a 3-month treatment period. In this study, galcanezumab resulted in a significantly greater decrease in the number of MMDs (average 4.1 days) compared to baseline 13.4 days versus placebo (average 1.0 day fewer compared to baseline 13.0 days) [97]. 

Regarding another endpoint, the change in acute headache medication use, the results of the CONQUER trial showed that the least-squares mean reduction was 4.2 in the verum group and 0.9 in the placebo group, demonstrating the potency of galcanezumab to reduce acute headache medication use (and overuse) [98]. 

Another important endpoint in the evaluation of the effect of a migraine prophylactic drug is the change in the rate of acute medication overuse within a certain period. A secondary analysis of the open-label extension of the REGAIN trial demonstrated that galcanezumab treatment significantly decreased the incidence of acute headache medication overuse (at month 3, the incidence was 33% in both dose groups of galcanezumab, compared to 46% in the placebo group; at month 12, it was 16% in the previous-galcanezumab 120 mg group and 23% in the previous-galcanezumab 240 mg group) [99].

The GARLIT trial (a 12-month, observational, longitudinal, cohort multi-center study), which evaluated the conversion from CM to EM, revealed that 52.3% of the CM patients showed consistent conversion to EM for the entire 12 month period [100]. 

The economic impact (direct and indirect costs) of a chronic disease for the sufferers is another important aspect of a drug treatment. Aiming to evaluate this aspect, a post-hoc analysis from multiple trials showed that employed CM patients had significant cost savings after galcanezumab treatment [101]. 

At the population level, galcanezumab demonstrated consistent efficacy in a 4-week-long treatment period in CM patients in a post-hoc analysis. The anecdotal phenomenon of the ‘wearing off’ of efficacy in the last week of the monthly galcanezumab treatment cycle was specifically assessed at the individual patient level in this analysis, revealing that the rates of patients meeting the threshold of ‘wearing off’ were low and similar among the placebo, galcanezumab 120 mg, and galcanezumab 240 mg treatment groups [102]. Of clinical interest, data provided by the retrospective pooled analysis of a small self-selected cohort from the completers of the open-label extension phases of studies with erenumab and galcanezumab in the preventive treatment of CM demonstrated long-term effects, with no significant difference in terms of the MMDs in the last 4 weeks of the open-label phase and the drug-free 12-week period after open-label treatment termination [103].

The combination of CGRP-targeting mAb therapy with other migraine prophylactic medications such BoNT-A represent a new potential therapeutic constellation. A real-world, retrospective, longitudinal study focusing on the safety and efficacy of CGRP-targeting mAbs added to BoNT-A in CM patients revealed that this drug combination was safe and well tolerated [104].

Overall, the SC monthly administration of galcanezumab is effective, safe, and well tolerated in CM patients, even in difficult-to-treat patients.

#### 4.2.4. Eptinezumab

Eptinezumab is a mAb (IgG1) targeted against the CGRP ligand, administered by IV for EM and CM patients.

The PROMISE-2 trial (a phase 3 RCT) was conducted to evaluate the efficacy and safety of eptinezumab treatment in CM patients. Based on the results, eptinezumab (100 mg and 300 mg) IV significantly decreased the baseline MMDs from day 1 of the administration through week 12 compared to the placebo in CM patients (placebo: −5.6; eptinezumab 100 mg: −7.7 (*p* value < 0.0001), and eptinezumab 300 mg: −8.2 (*p* value < 0.0001). The most commonly reported AE was nasopharyngitis (>2% of treated patients) [68]. A 24-week-long extension of the PROMISE-2 study revealed that both doses provided sustained beneficial effects (i.e., in the reduction of MMDs, in the increase in ≥50% and ≥75% migraine responder rates, and in the improvements in the patient rates in terms of the HIT-6 and the Patient Global Impression of Change scores) [105].

A subgroup analysis of the PROMISE-2 trial focusing on patients with a dual diagnosis of CM and MOH evaluated the effect of eptinezumab (100 mg and 300 mg) compared to the placebo. The results showed that, compared to the average 16.7 MMDs at baseline, eptinezumab-treated patients experienced significantly greater decreases in MMDs (−8.4 for eptinezumab 100 mg and −8.6 for 300 mg) than those treated with the placebo (−5.4) over the 12 weeks of treatment [106].

A post-hoc analysis of the PROMISE-2 study evaluated the reduction of acute headache rescue medication use in patients suffering from both CM and MOH. The findings showed that the total monthly acute headache medication use decreased from 20.6 days per month at baseline to 10.6 days per month over the 24 weeks of eptinezumab 100 mg treatment, while eptinezumab 300 mg treatment resulted in a decrease from 20.7 to 10.5 days per month (compared to the observed decrease with the placebo from 19.8 to 14.0 days per month) [107]. A post-hoc analysis of the PROMISE-2 study, which aimed to evaluate the early response to eptinezumab in CM during the first one-month period showed that the ≥50% treatment responder rate was 54.5% in the eptinezumab 100 mg group, 60.6% in the eptinezumab 300 mg group, and 36.1% in the placebo group [108]. Another post-hoc analysis of the PROMISE-2 study aimed to evaluate the reduction in MMDs in CM patients older than 50 years of age. The results showed that the mean changes in MMDs were −7.7 and −8.6 with eptinezumab 100 mg and 300 mg, respectively, versus −6.0 with the placebo. It was also concluded that the eptinezumab treatment was tolerable and safe in this group of elderly CM patients [109].

Yet another post-hoc subgroup analysis of the PROMISE-2 study, which focused on CM patients with self-reported history of aura, concluded that the number of MMDs changed by −7.1 with eptinezumab 100 mg and −7.6 with eptinezumab 300 mg versus −6.0 with the placebo over weeks 1–12, and these results are comparable to those in the overall study population [110].

A recent post-hoc analysis of the PROMISE-2 trial measuring the dose-related efficacy of eptinezumab in CM patients over weeks 1–12 and weeks 13–24 revealed a similar likelihood of achieving a ≥50% migraine responder rate for either dose (i.e., 100 mg or 300 mg) [111]. An analysis evaluated the patient-identified most bothersome symptom (such as light sensitivity, nausea/vomiting, and pain aggravated by activity) in CM patients from the PROMISE-2 study. The magnitude of the standardized mean differences between the placebo and treatment groups was 0.31 (100 mg versus placebo) and 0.54 (300 mg versus placebo) [112]. The migraine-associated burden was also analyzed based on the PROMISE-2 trial focusing on the mean MHDs and mean monthly headache episodes (migraine attacks). The reduction in the mean MHDs was 8.9 (eptinezumab 100 mg) and 9.7 (eptinezumab 300 mg) versus 7.3 in the placebo over the first 4-week period. This beneficial effect of eptinezumab was maintained throughout the 24 weeks until the end of the study period. The mean monthly headache episodes decreased by 8.4 (eptinezumab 100 mg) and 9.0 (eptinezumab 300 mg) versus by 7.1 with the placebo [113]. A sub-analysis of PROMISE-2 addressing quantity changes in acute headache medication use in a subgroup of CM with MOH patients with ≥50% response (over weeks 1–24) revealed that the proportion of days with headache and triptan use decreased by 14.5% with eptinezumab compared to 12.6% with the placebo [114].

The DELIVER trial, a multi-center, multi-arm, phase 3b study comprising a 24-week double-blind, a placebo-controlled period and a 48-week dose-blinded extension, which investigated CM patients with two to four previous preventive treatment failures revealed that the change from baseline to weeks 1–12 in mean MMDs was −4.8 with eptinezumab 100 mg and −5.3 with eptinezumab 300 mg compared to −2.1 with the placebo [115]. The PREVAIL study, which was a long-term (2-year), open-label, phase 3 trial addressing the safety and tolerability of eptinezumab (300 mg by 30-min IV administration every 12 weeks for up to 8 doses) in patients with CM demonstrated good safety and tolerability, an early and sustained decrease in migraine-related burden, and an improvement in the health-related quality of life. The notable AEs were nasopharyngitis (14.1%), upper respiratory tract infection (7.8%), and sinusitis (7.8%) [116].

The post-hoc analysis of the PREVAIL study aimed to investigate the changes in the MIDAS questionnaire over 2 years in eptinezumab-treated CM patients. The mean MIDAS scores evaluating the absenteeism related to missed work/school, household work, and family/social/leisure activities changed from 9.7 days at baseline to 3.2 days at week 12 and to 3.9 days at week 104, whereas those measuring the presenteeism of reduced productivity in work/school or household work changed from 14.2 days at baseline to 5.2 days at weeks 12 and 104 [117].

A recent meta-analysis concluded that eptinezumab in doses of 100 mg and 300 mg showed significant efficacy for the prevention of migraines (analyzing EM and CM trials together). Eptinezumab reduces the MMDs, increases the 75% and 50% migraine responder rates, and decreases the HIT-6 score both on week 4 and week 12. Eptinezumab showed similar incidence AEs compared to the placebo [118].

Overall, eptinezumab is the only CGRP-targeting mAb which is administered by IV and has a real fast effect on day 1 after dosing. Eptinezumab is effective, well tolerated, and safe in preventive treatment of CM.

### 4.3. Different Types of Anti-CGRP mAbs in Drug-Resistant CM Patients

A network meta-analysis of RCTs compared the efficacy and tolerability of CGRP-targeting mAbs with BoNT-A or topiramate for CM. The results revealed that all four CGRP mAbs (i.e., eptinezumab, erenumab, fremanezumab, and galcanezumab) have outstanding safety, tolerability, and efficacy compared to BoNT-A and topiramate conventional preventive medications for CM [119].

The recommended mAb treatment duration is between 6 and 12 months, and the condition of CM patients after treatment discontinuation is still unclear. A single-center, prospective, cohort study addressed this question, following-up on drug-refractory CM patients (all with MOH) who were treated with erenumab or galcanezumab for 12 months, discontinued the treatment for 3 months, and restarted again for 1 month. They observed that three-quarters of the patients reported a worsening of their condition in the discontinuation phase. This subgroup of patients required retreatment. One-quarter of CM patients, who had better quality-of-life scores at baseline (based on MIDAS and HIT-6 test), had sustained benefit and did not need retreatment. Predicting the state of CM patients after the discontinuation of mAb treatment is always challenging. Based on this small subject number trial, better quality-of-life scores before treatment initiation may predict a sustained treatment response [120].

An exploratory, prospective, questionnaire-based study evaluating the effectiveness of anti-CGRP mAbs (erenumab, fremanezumab, and galcanezumab) on migraine prodromal and accompanying symptoms in CM patients revealed that only 8.8% of patients experienced photophobia, 5.0% phonophobia, 6.3% osmophobia, 7.5% nausea, and 1.3% vomiting. These results highlight that this treatment is likely to prevent not only the head pain component of migraines but also the migraine-associated symptoms [121].

In the case of difficult-to-treat pharmacoresistant CM patients with or without medication overuse, treatment with classical prophylactic drugs is usually unsuccessful. A single-center, prospective, cohort study focusing on the long-term (12-month) effectiveness of three anti-CGRP mAbs (erenumab, galcanezumab and fremanezumab) in this special subgroup of CM patients revealed that the MIDAS score was reduced at all follow-ups compared to baseline (≥50% reduction in MIDAS score was reported by 84.4–100% of CM sufferers) [122]. A meta-analysis revealed that CGRP-targeted mAbs had a higher odds ratio in achieving the ≥50% response rate versus the placebo in the preventive treatment of CM [42].

A large, multicenter, cohort, real-life study evaluating the ≥50% response predictors at 24 weeks to the SC administered mAbs (erenumab, galcanezumab, or fremanezumab) revealed that in CM, the ≥50% response was positively associated with unilateral cranial autonomic symptoms, unilateral pain plus unilateral autonomic symptoms, and unilateral pain with allodynia, whereas it was negatively associated with obesity [123]. Real-world evidence from the analysis of clinical routine data of highly resistant EM and CM patients after a mandatory break were followed by 2 years following a 9–12-month treatment with anti-CGRP mAbs (erenumab, galcanezumab, and fremanezumab) revealed that the mean number of MMDs significantly increased (by 5.06 days) during the treatment break in CM patients [124]. A recent pooled analysis of real-world evidence demonstrated for the first time that the combined treatment with anti-CGRP mAbs (erenumab, fremanezumab, and galcanezumab) (−1.97 MHDs compared to baseline) plus onabotulinumtoxinA leads to a 2.67-day reduction in MHDs compared to onabotulinumtoxinA administration alone in CM patients (where anti-CGRP mAbs or onabotulinumtoxinA alone resulted in reductions of 1.94 and 1.86 MHDs compared to baseline, respectively) [125]. It is a well-known phenomenon that in chronic pain conditions, the medications for pain relief have a high placebo effect. It is a question whether this observation is influenced by the route of administration of the effective drug. A meta-analysis evaluating placebo responses in CM patients without comorbidities compared patients receiving placebo agents by SC, IV, or oral routes of administration to those who received multiple head injections. The placebo effect of multiple head injections (i.e., in BoNT-A studies) was superior to other routes such as IV (i.e., in eptinezumab studies), SC (i.e., in erenumab, fremanezumab, and galcanezumab studies), and oral (i.e., in topiramate studies) [126].

In summary, all doses of CGRP-targeting mAbs are efficacious, safe, and well tolerated in the prophylactic therapy of CM patients, predominantly in highly resistant, difficult-to-treat cases, based on the results of RCTs, meta-analyses, and data from long-term real-world settings [127,128].

### 4.4. Neuromodulation for Drug-Refractory CM

Neuromodulatory methods are one of the emerging non-pharmacological therapeutic options for difficult-to-treat, drug-resistant or drug-refractory CM cases. Based on the European Headache Federation (EHF) consensus, “resistant migraine is defined by having failed at least 3 classes of migraine prophylactic drugs and suffer from at least 8 debilitating headache days per month for at least 3 consecutive months without improvement. While refractory migraine defined by having failed all of the available preventatives and suffer from at least 8 debilitating headache days per month at least 6 consecutive months” [129]. These techniques include peripheral ganglia and nerve blockades as well as neurostimulation of the peripheral and central nervous systems [23,130,131]. 

#### 4.4.1. Peripheral Ganglia and Nerve Blockades

A recent meta-analysis addressing the efficacy of chemical blockade (i.e., with a local anesthetic) of the greater occipital nerve in CM patients concluded that this method reduced both the frequency and intensity of headache compared to the placebo [132].

A pilot RCT study examining repetitive sphenopalatine ganglion blockade with the Tx360 device with bupivacaine (0.5%) compared to salina demonstrated significantly decreased headache intensity in intractable CM patients. Later, the long-term observation also showed a beneficial effect [133,134].

#### 4.4.2. Neurostimulation

##### Peripheral Neurostimulation

(1) Non-Invasive Peripheral Neurostimulation

(1.1) Transcutaneous Cervical Vagus Nerve Stimulation (VNS) (the Gamacore Device)

The EVENT study revealed that, at 2 months, 9.1% of the CM patients achieved more than 50% treatment response at the double-blind condition, whereas in the open-label phase at 8 months, the response rate was 46.7% [135].

(1.2) Transcutaneous Auricular VNS (the NEMOS Device)

A RCT demonstrated that in a 1 Hz-stimulated group of intractable CM patients had 50% or greater reduction in MHDs in 29.4% of the patients, while in the 25 Hz group, it was only 13.3% [136].

(1.3) Remote Electrical Neuromodulation (REN)

It is a recently developed neuromodulatory technique that applys a wireless, wearable, non-invasive device controlled by a smartphone application, which stimulates the peripheral nerves of the upper arm. It is an effective, well tolerated, and safe non-pharmacological method for acute treatment in CM [137,138,139].

(2) Invasive Peripheral Neurostimulation

(2.1) Implantable Occipital Nerve Stimulation (ONS)

A long-term RCT revealed that surgically implanted ONS in intractable CM patients significantly decreased the numbers of MHDs by 7.7 (±8.7)% days.

In intractable CM, regarding peripheral neuromodulatory methods, transcutaneous cervical vagal nerve stimulation (VNS) and greater occipital nerve blockade (with lidocaine or bupivacaine) might be offered as the first choice (being non-invasive), whereas invasive (i.e., implantable) techniques such as occipital nerve stimulation (ONS), combined supraorbital and ONS, and transcutaneous auricular VNS can be offered as a second choice [131].

##### Central Neurostimulation

(a) Non-Invasive Central Neurostimulation

High-frequency repetitive transcranial magnetic stimulation (rTMS) seemed to have positive effects (on pain severity and headache frequency) in intractable CM in a recent meta-analysis.

(a.1) Transcranial Magnetic Stimulation (TMS)

An early pilot clinical trial revealed that repetitive transcranial magnetic stimulation (rTMS) of the dorsolateral prefrontal cortex (DLPFC) resulted in a lower frequency of migraine attacks and number of rescue medication compared to sham group [140]. A randomized proof-of-principle trial using high-frequency rTMS concluded that the motor cortex stimulation is a more promising region than the DLFPC [141].

Neuronavigation functional MRI-based 10-min-long sessions (given 5 days per week over 2 consecutive weeks) of rTMS (600 pulses in 10 trains and 10 Hz with an intertrain interval of 60 sec) over the left motor cortex in right-handed CM patients showed a significant reduction in the mean VAS rating, headache frequency, and MIDAS questionnaire in the real rTMS group compared to the sham group [142].

A long-term, prospective, single-center, open-label, real-world study using single-pulse TMS targeting the base of the skull in patients with difficult-to-treat CM with or without medication overuse headache (MOH) concluded that this method may be effective and well tolerated for the long-term prevention of CM [143].

(a.2) Transcranial Direct Current Stimulation (tDCS)

A long-term RCT examining the effect of tDCS in CM with MOH patients concluded that there was no efficacy in the 50% reduction in MHDs at 12 months [144]. A recent RCT comparing one vs. two daily sessions of anodal tDCS delivered to the left DLPFC in female CM patients revealed a significant decrease in migraine-related disability in the one daily tDCS session [145]. 

Different neuromodulatory methods can be beneficial for selected pharmacoresistant CM patients.

## 5. Psychological Therapeutic Options in CM

Migraines have a wide range of comorbidities such as vascular, cardiac, neurological, psychiatric, and miscellaneous (e.g., snoring, allergy/asthma). Psychiatric conditions related to migraines are the following: depression, bipolar affective disorders, generalized anxiety, and somatoform disorders. The mentioned ones seem to be more common in women compared to men, and more frequent in CM patients than EM patients [146,147]. Based on epidemiological data, the lifetime prevalence of major depression is three times higher in migraine patients than in non-migraineurs [146]. Comorbidities, especially psychiatric ones together with other risk factors for migraine chronification, influence the clinical course of CM and quality of life of the patients. Both non-modifiable (e.g., female gender, age, low education) and modifiable (e.g., stressful life events, medication overuse, sleep disturbances, obesity) risk factors have fundamental roles in transforming EM to chronic form [148]. Recognizing the lifestyle factors which have a crucial role in the above process is the first step for the non-pharmacological psychological treatment of CM patients. Psychological treatment can be a beneficial adjuvant treatment to pharmacological therapies. Treating chronic pain, like CM multimodal management is recommended, and behavioral, physical, and pharmacological treatment should be integrated [149]. Behavioral therapies, namely relaxation, biofeedback, cognitive behavioral therapy (CBT), and mindfulness-based cognitive therapy, are helpful methods for minimizing the role of risk factors in CM patients. A phase 3 RCT comparing the feasibility and short-term efficacy of mindfulness added to treatment as usual and usual treatment alone for CM patients indicated that adding mindfulness to treatment as usual was feasible with short-term efficacy versus treatment without mindfulness [150]. A clinical study aimed at examining neuropsychological and neuropsychiatric characteristics of CM patients during the interictal phase concluded that CM patients showed a higher rate compared to healthy controls in cognitive domains (such as sustained attention, information processing speed, visuospatial episodic memory, working memory, and verbal fluency) and depressive and anxiety symptoms [151]. In a case of CM, the integrated model and the pharmacological options together present beneficial management of the patients.

## 6. Discussion

Adequate classification of diseases is the fundament of the development of diagnostic criteria and evidence-based treatments. Healthcare professionals are expected to be familiar with these systems in their field in detail. Certain diseases belong to more than one classification system, and CM is a typical example for this phenomenon. Chronic primary pain syndrome is per se a disease in its own right, whereas CM is a primary headache disorder. As per the multiple parenting concept, however, CM is a chronic primary pain syndrome as well. Therefore, as a dual diagnosis and as a *Janus* face, CM exhibits features of both a headache disorder and of a chronic pain condition. In everyday clinical practice, this means that a distinct disease harbors characteristics of both diagnostic criteria. For example, in CM as a chronic primary pain syndrome, one of the common clinical signs may be allodynia, a feature typical of pain with a neuropathic component. The prevalence of cutaneous allodynia in patients with migraine is rather high, estimated to be between 40–70%. The presence of allodynia is a marker of central sensitization in migraineurs and is associated with an elevated risk for chronification [152]. In addition, with its dual origin from two distinct arms of diseases (i.e., chronic pain syndromes and primary headache disorders), CM is a highly prevalent condition, having a great socio-economic impact and posing an individual burden. Patients suffering from CM are in need of effective treatment. Findings extracted from basic science by means of translational medicine are cornerstones in the development of evidence-based pharmacological therapy. The origo of such a concept in CM is CGRP, with its proven effects on the TS. Via the CGRP pathway, BoNT-A and CGRP-targeting mAbs can influence the hyperactivation of the TS. Favorable preclinical data, however, only mean a treasure in the hand of clinicians if they work in well-designed clinical trials as well as in real-life clinical practice. In the cases of both BoNT-A and CGRP-targeting mAbs, the beneficial effects in CM have been confirmed.

In line with all these, the therapeutic guideline of the EHF-recommended BoNT-A (i.e., onabotulinumtoxinA) is an effective and well-tolerated treatment of CM [41]. The latest update of the EHF therapeutic guideline recommended the CGRP-targeting mAbs (erenumab, fremanezumab, galcanezumab, and eptinezumab) as the first-line treatment options in migraine prophylactic treatment (for both EM and CM), recognizing them as effective and safe also in the long term [69].

## Figures and Tables

**Table 1:** Classification of migraine [2].

1.1 Migraine without aura
1.2 Migraine with aura
1.2.1 Migraine with typical aura
1.2.2 Migraine with brainstem aura
1.2.3 Hemiplegic migraine
1.2.4 Retinal migraine
1.3 Chronic migraine
1.4 Complications of migraine
1.4.1 Status migrainosus
1.4.2 Persistent aura without infarction
1.4.3 Migrainous infarction
1.4.4 Migraine aura-triggered seizure
1.5 Probable migraine
1.5.1 Probable migraine without aura
1.5.2 Probable migraine with aura
1.6 Episodic syndromes that may be associated with migraine
1.6.1 Recurrent gastrointestinal disturbance
1.6.2 Benign paroxysmal vertigo
1.6.3 Benign paroxysmal torticollis

**Table 2:** Classification of chronic pain [1,3].

Chronic Pain	
Chronic Primary Pain	Chronic Secondary Pain
Chronic widespread pain	Chronic cancer-related pain
Complex regional pain syndrome	Chronic postsurgical or posttraumatic pain
	Chronic neuropathic pain
Chronic primary headache or orofacial pain	Chronic secondary headache or orofacial pain
Chronic primary visceral pain	Chronic secondary visceral pain
Chronic primary musculoskeletal pain	Chronic secondary musculoskeletal pain

**Table 3:** Diagnostic criteria of chronic primary pain [3].

A. Chronic pain (persistent or recurrent for longer than 3 months) is present
B. The pain is associated with at least one of the following aspects:
B.1 Significant emotional distress (e.g., anxiety, anger, frustration, or depression)
B.2 Significant functional disability (interference with daily life activities and social participation)
C. The pain is not better accounted for by another chronic pain condition

**Table 4:** Diagnostic criteria of chronic migraine [2].

A. Headache (migraine-like or tension-type-like) on ≥15 days/month for >3 consecutive months, and fulfilling criteria B and C
B. Occurring in a patient who has had at least five attacks, fulfilling criteria C–D for *migraine without aura* and/or criteria B–C for *migraine with aura*
C. On ≥8 days/month for >3 months, fulfilling any of the following:
1. criteria C and D for *migraine without aura*
2. criteria B and C for *migraine with aura*
3. believed by the patient to be migraine at onset and relieved by a triptan or ergot derivative
D. Not better accounted for by another ICHD-3 diagnosis

ICHD-3: International Classification of Headache Disorders, 3rd edition.

**Table 5:** Data from the pooled PREEMPT clinical program.

Substance	Site of Action	Mode of Action	Route of Administration	Dosing	Indication	Numbers of Participants		Results	*p* Value	Adverse Events	Ref.
botulinumtoxin type A (BoNT-A)	SNARE complex	inhibition of SNAP-25, modulates peripheral and central sensitization	multiple cranial and cervical injections (fixed sites)	155–195 units (fixed dose) every 12 weeks	chronic migraine	BoNT-A *n* = 688	placebo *n* = 696	mean decrease from baseline in frequency of headache days at week 24 BoNT-A: −8.4 days placebo: −6.6 days	<0.001	neck pain (6.7%), localized muscular weakness (5.5%), eyelid ptosis (3.3%)	[33]

BoNT-A: botulinum toxin type A; PREEMPT: the Phase III Research Evaluating Migraine Prophylaxis Therapy; SNAP-25: synaptosomal-associated protein of 25 kDa; SNARE: soluble N-ethylmaleimide-sensitive factor attachment protein.

**Table 6:** Characteristics of the recommended prophylactic drug treatment for chronic migraine: CGRP-targeting mAbs.

Substance	Type of IgG	Site of Action	Mode of Action	Route of Administration	Dosing	Indication	Numbers of Participants		Results		*p* Value	Adverse Events	Ref.
eptinezumab	fully humanized IgG1	CGRP ligand	selectively binds to CGRP isoforms	IV	100 mg or 300 mg (a single dose through week 12)	EM and CM	eptinezumab 100 mg *n* = 356; eptinezumab 300 mg *n*= 350	placebo *n* = 366	reduction in MMDs: 100 mg −7,7; placebo −5.6	reduction in MMDs: 300 mg −8.2, placebo −5.6	*p* < 0.0001	nasopharyngitis (>2%)	[68,69]
erenumab	human IgG2	CGRP receptor	competitively and reversibly binds to CGRP-receptor	SC	70 mg or 140 mg monthly	EM and CM	erenumab 70 mg *n* = 191; erenumab 140 mg *n* = 190	placebo *n* = 286	reduction in MMDs in both (100 mg and 300 mg) doses: −6.6 days versus placebo −4.2 days		*p* < 0.0001	nasopharyngitis (70 mg 3%; 140 mg 2%; placebo 6%); constipation (70 mg 0%; 140 mg 4%; placebo 1%); injection site pain (70 mg 4%; 140 mg 4%; placebo 1%)	[69,70]
fremanezumab	fully humanized IgG2A	CGRP ligand	selectively binds to CGRP isoforms	SC	675 mg quaterly or 225 mg monthly	EM and CM	fremanezumab quarterly *n* = 376; fremanezumab monthly *n* = 379	placebo *n* = 375	the least-squares mean reductions in the average number of MHDs: 4.3 ± 0.3 quarterly; 4.6 ± 0.3 monthly; 2.5 ± 0.3 placebo ±		*p* < 0.001	nasopharyngitis (quarterly 5%, monthly 4%, placebo 5%), injection site pain (quarterly 30%, monthly 26%, placebo 28%)	[69,71]
galcanezumab	fully humanized IgG4	CGRP ligand	binds to CGRP as a ligand Inhibits its biological activity	SC	120 mg monthly (with loading dose of 240 mg) or 240 mg monthly	EM and CM	galcanezumab 120 mg *n* = 278; galcanezumab 240 mg *n* = 277	placebo *n* = 558	the mean reduction in the number of MHDs: 120 mg −4.8 days; 240 mg −4.6 days; placebo −2.7 days		*p* < 0.001	nasopharyngitis (120 mg 6%; 240 mg 3%; placebo 5%), injection site pain (120 mg 6%; 240 mg 7%; placebo 4%)	[69,72]

CGRP: calcitonin gene-related peptide; CM: chronic migraine; EM: episodic migraine; IgG: immunoglobulin G; IV: intravenous; MHD: monthly headache day; MMD: monthly migraine day; mAbs: monoclonal antibodies; SC: subcutaneous.

## Data Availability

This work is a review. The data used to prepare this work are available in the cited sources.

## Abbreviations

AE	adverse event
BBB	blood-brain barrier
BoNT-A	botulinumtoxin type A
CGRP	calcitonin gene-related peptide
CM	chronic migraine
EM	episodic migraine
EHF	European Headache Federation
IASP	International Association for the Study of Pain
ICHD-3	International Classification of Headache Disorders, 3rd edition
IV	intravenous(ly)
MIDAS	Migraine Disability Assessment
MHD	monthly headache day
MMD	monthly migraine day
mAb	monoclonal antibody
ONS	occipital nerve stimulation
RCT	randomized controlled trial
rTMS	repetitive transcranial magnetic stimulation
SC	subcutaneous(ly)
SNARE	soluble N-ethylmaleimide-sensitive factor attachment protein
SNAP-25	synaptosomal-associated protein of 25 kDa
TS	trigeminovascular system
VNS	vagal nerve stimulation
